# Embedded Ultrasonic Transducers for Active and Passive Concrete Monitoring

**DOI:** 10.3390/s150509756

**Published:** 2015-04-27

**Authors:** Ernst Niederleithinger, Julia Wolf, Frank Mielentz, Herbert Wiggenhauser, Stephan Pirskawetz

**Affiliations:** BAM Federal Institute for Materials Research and Testing, Berlin 12200, Germany; E-Mails: julia.wolf@bam.de (J.W.); frank.mielentz@bam.de (F.M.); herbert.wiggenhauser@bam.de (H.W.); stephan.pirskawetz@bam.de (S.P.)

**Keywords:** ultrasound, transmission, concrete, damages, cracks, stress

## Abstract

Recently developed new transducers for ultrasonic transmission, which can be embedded right into concrete, are now used for non-destructive permanent monitoring of concrete. They can be installed during construction or thereafter. Large volumes of concrete can be monitored for changes of material properties by a limited number of transducers. The transducer design, the main properties as well as installation procedures are presented. It is shown that compressional waves with a central frequency of 62 kHz are mainly generated around the transducer’s axis. The transducer can be used as a transmitter or receiver. Application examples demonstrate that the transducers can be used to monitor concrete conditions parameters (stress, temperature, …) as well as damages in an early state or the detection of acoustic events (e.g., crack opening). Besides application in civil engineering our setups can also be used for model studies in geosciences.

## 1. Introduction

Concrete is a complex, multi-phase material. It is made of hydraulic cement, water and aggregates in lots of variations. The first two ingredients start to hydrate and crystallize when in contact with each other. This process is fast in the first hours and days, but can continue for months or years. The aggregates, gravel or crushed stone of various types and sizes (µm-cm range), are used as a filler to save cost and energy. Concrete contains pores, either filled with unbound water or air.

Concrete is the material most produced by mankind. It is considered to be strong, resistive and durable. Some early concrete structures as the cupola of the Roman Pantheon are standing tall after almost 2000 years. However, under certain conditions (hostile environments, adverse load conditions) concrete constructions require attention. For example the apparently ever increasing traffic load (number and individual load of trucks) on bridges may lead to deterioration much earlier than expected at the time of design. Currently, inspections are still mainly based on visual methods, but sophisticated non-destructive methods are used more and more often. The use of bridge instrumentation increases rapidly [1]. However, monitoring is so far limited to local sensors (e.g., strain gauges), which are probing just their close vicinity, or global methods as modal analysis, which looks to the structure as a whole. There is a gap in between. A method which would look at a certain critical volume of concrete with a very limited number of sensors would be of great value.

Ultrasonic transducers with frequencies from 25 kHz to 400 kHz have been used for concrete since decades. They are used in the lab on samples (and sometimes on site) in transmission mode to measure elastic properties and to assess degradation. New point contact transducers have revolutionized the use of echo techniques for thickness measurements and structural imaging. Even the detection of voids in tendon ducts seems to be possible [2]. All transducers used in practice today are for surface mounting. For monitoring this approach shows three strong disadvantages. First, the need for constant coupling, which is hard to realize on the surface in practice. Second, the high influence of surface and external effects (temperature and others) leads to unwanted effects on the measurements. Third, in practical field applications the transducers are prone for accidents or vandalism. Therefore we started to develop a novel transducer, which can be permanently embedded in concrete.

This is not the first or only attempt to embed ultrasonic transducers in concrete. Similar ideas have been proposed e.g., by [3,4], but only in experimental setups for lab applications. For practical applications a much more robust approach would be required. An experiment conducted more than 35 years ago to monitor the hardening of concrete at a massive water dam in Saxony, Germany, by embedded ultrasonic transmitters and receivers recently gave us the opportunity to prove that this kind of sensors might survive in concrete for decades [5].

## 2. A novel Ultrasonic Transducer to Be Embedded in Concrete

### 2.1. Transducer Design and Description

**Figure 1:**
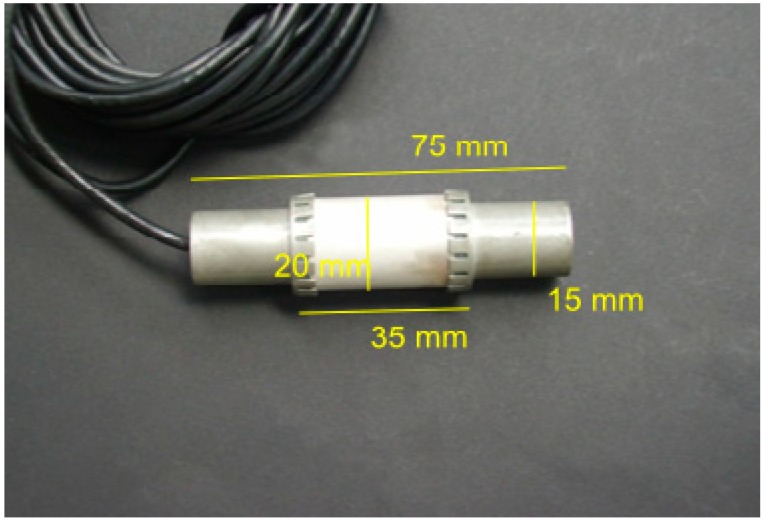
Photograph of the novel ultrasonic transducer for embedment in concrete (Manufacturer: Acsys Ltd., Moscow, Russia. Photo: BAM).

New ultrasonic transducers (“SO807”) have been designed by Acoustic Control Systems, Ltd. (ACS, Moscow, Russia) in cooperation with and exclusively for BAM (Figure 1). The main part is a hollow piezoceramic cylinder of 20 mm diameter and 35 mm length. The electric connections are on the inside. On both ends metallic pieces are clamped to the piezoceramic part. The outer diameter of 15 mm allows stacking of several transducers along a line using standard PVC tubes. Having all cables inside ensures good coupling of the piezo to the concrete and protects the electrical connections during installation. The total length of the transducer is 75 mm.

### 2.2. Installation

**Figure 2:**
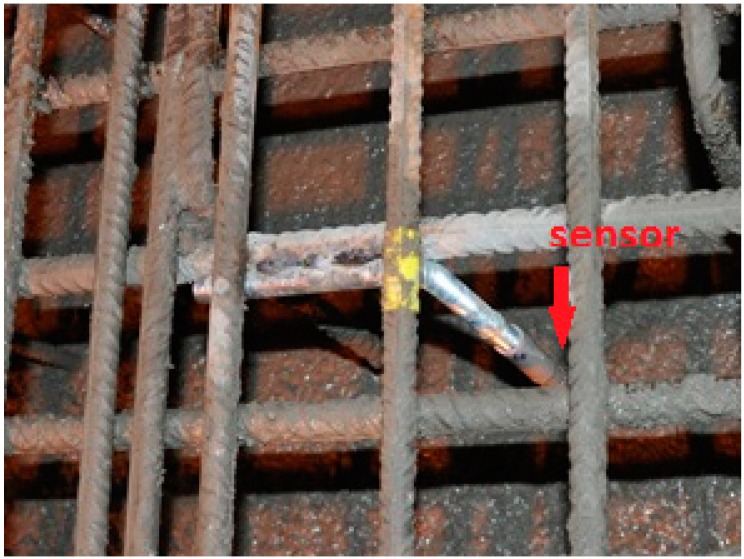
Photograph of an ultrasonic transducer mounted at a bridge construction site before casting (Photo: Neostrain S.A.).

The transducers can easily be installed at the time of construction. To ensure that they keep their position during casting and vibration, they have to be mounted either direct to the reinforcement or using some kind of stabilizing construction. In our latest bridge installation we have used L-shaped pieces of rebar welded to the reinforcement to hold the transducers at the specified position (Figure 2). Another possibility would be to mount stacked series of transducers using PVC tube segments (similar to the method used for installation in existing structures described below).

**Figure 3:**
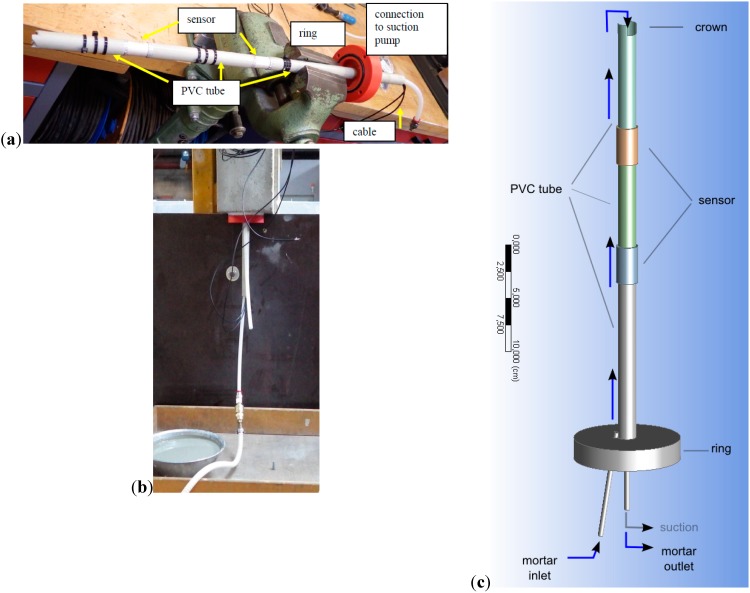
(**a**): Two transducers connected by PVC tube segments and equipped with sealing cap for post-concreting installation; (**b**): Installation into a concrete specimen; (**c**): Sketch of grout flow during installation.

For existing structures we have developed a method to install one or several transducers at the required depth(s) in a drill hole and ensuring sufficient coupling to the concrete. The hole is drilled with a slightly larger diameter than the transducers and slightly deeper than the installation depth. The transducer(s) and the tube segments are connected with a sealing cap to the structures’ surface (Figure 3). The cap contains an inlet, which is connected to a (liquid) grout reservoir and to the space between the transducers/tubes and the concrete. By connecting a suction pump via an outlet in the cap to the inner hollow space of the transducers and the tubes the grout is sucked into the drill hole and via the crown of the tubes into the inner space (Figure 3c). When the grout appears at the outlet we can be sure the entire space is (at least almost) filled and the transducers are coupled to the concrete. We are using a fast hardening, slightly expanding type of grout.

### 2.3. Characterization

**Figure 4:**
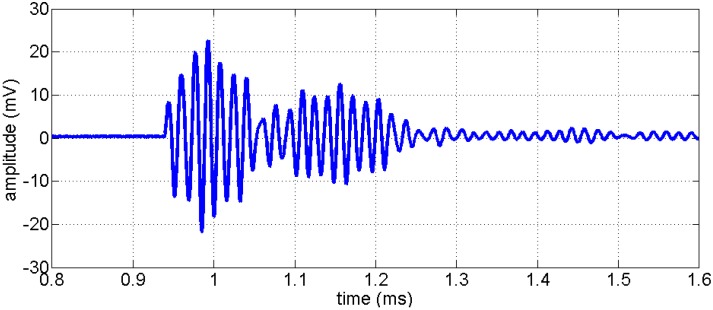
Response of the novel transducer to a short electrical impulse (2 µs), recorded by a second, identical one mounted back to back. Time of onset due to recording hardware settings.

The transducers have been characterized by some basic experiments. First, two transducers of the same type have been installed back to back directly without any medium in between to evaluate the frequency spectrum. A short impulse (t = 2 µs) was used as input. The signal recorded by the transducer is displayed in Figure 4. The signal recorded shows a wavelet with about 20 periods and a total duration of ca. 0.3 ms. The reverberations show a lack of damping of the piezo material. As we don’t intend to use the transducers for imaging applications, where a sharp response (broadband in frequency domain) would be more beneficial, this behavior is fully satisfactory.

**Figure 5:**
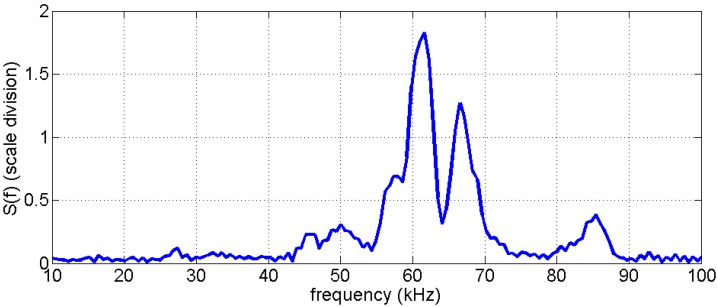
Amplitude spectrum of data shown in Figure 4.

The amplitude spectrum of the data of Figure 4 is shown in Figure 5. There is a prominent frequency peak at 62 kHz and a significant second one at 65 kHz. Smaller peaks appear around 50 and 85 kHz. There is no significant energy with frequencies lower than 40 or higher than 90 kHz.

A frequency of 62 kHz relates to a wavelength in concrete (compressional wave speed of about 4000 m/s) of ca. 65 mm. This is at least double the size of most aggregates (max aggregate size 32 mm in many types of concrete).

**Figure 6:**
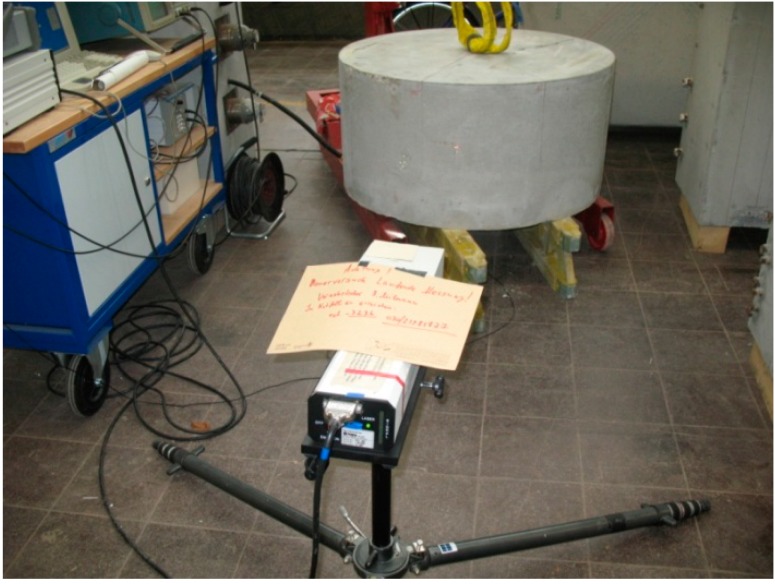
Experimental setup for measurement of directivity patterns. Transmitting transducers are embedded in the center of the concrete block. Surface movements are recorded by a laser vibrometer (in front).

To evaluate the directivity pattern of the new transducer, we have conducted two experiments, the first in a water pool, the second using transducers embedded horizontally or vertically, respectively, in two separate cylindrical concrete blocks (Figure 6). In the first case (water pool) an identical transducer (vertically oriented) was used as receiver. In the second experiment a laser vibrometer was used to record the surface vibrations of the concrete. The laser vibrometer’s position was fixed while the block was rotated in 10° intervals before each excitation. In both cases the measurements were taken using (a) vertical and (b) horizontal orientation of the transmitting transducers.

**Figure 7:**
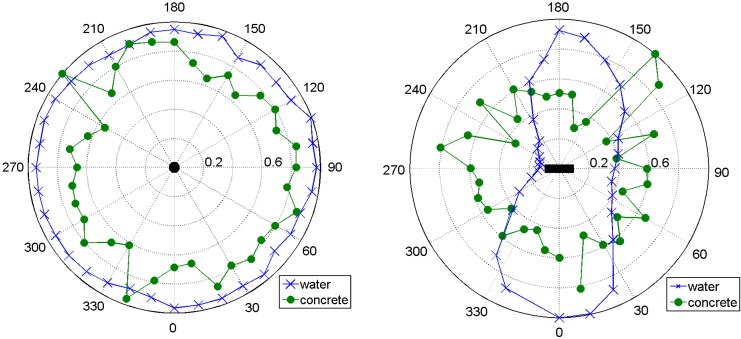
Directivity patterns for the new transducer, measured in water (blue crosses) and concrete bodies (green dots). (**Left**) vertical orientation; (**Right**) horizontal orientation. Axis from center to perimeter: relative amplitude. Seen from above.

The results of the directivity measurements are shown in Figure 7. To produce these plots, the amplitude of the direct arrivals (maximum of the first cycle) have been taken in 10° steps and normalized to the maximum value of all of them. For a vertical transmitter (right in Figure 7) we have recorded an almost perfect circle in water. This had to be expected as the transmitter is rotational symmetric round its vertical axis. In concrete the circular radiation pattern is still recognizable, but far from being perfect, probably due to the inherent inhomogeneities in concrete.

For horizontal transmitter orientation (left in Figure 7) the directivity pattern in water resembles something close to a number eight. Apparently (and not unexpectedly as applying a voltage to the cylindrical piezoceramic leads mainly to changes in diameter, not in length) the amplitudes emitted in the direction of the transducer’s rotational axis are much smaller than in the perpendicular direction. The experiment with the same configuration in concrete showed that the amplitude is similar in all directions (but has significant variation). Our interpretation is that due to scattering at the concrete’s aggregates, pores and cracks, the directivity pattern is somewhat equalized.

**Figure 8:**
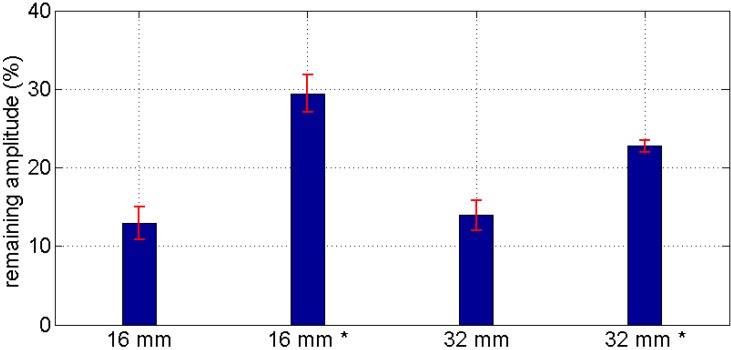
Amplitude ratios between near and far receivers in the attenuation experiment for the two specimens (16 and 32 mm max. aggregate size). The * marks values for transducers installed after concreting.

Additional experiments have been performed to get an idea on the range of our new transducers. This was done to assess the maximum possible distance between transmitters and receivers in future sensor networks in massive concrete structures. For this purpose a set of transducers was installed in two concrete blocks partly with, partly without reinforcement. Some transducers were installed before casting, some afterwards (see Section 2.2). One block had a maximum aggregate size of 16 mm, the other 32 mm. For all scenarios (aggregate size, reinforcement, and installation type) we have used the same measurement setup. One of the transducers, excited with a 100 V square pulse, was used as transmitter. At two others (distance *r* = 0.25 and 0.75 m, respectively, from the transmitter) the amplitudes A (maximum of first cycle) of the first arrivals were recorded. The amplitude ratios are shown in Figure 8. It shows, that the attenuation is mainly dependent on the installation type, but not on aggregate size or reinforcement (the latter not shown here). The applicable range of our transducers was estimated from these data to be around three meters [6,7]. For this we calculated the material and frequency specific damping constant α from the amplitudes (A_1_, A_2_) of the first arrival after r_1_ = 0.25 m and r_2_ = 0.75 m using:

(1) $$-\text{α}=\left[\ln\left(\frac{A_{2}}{A_{1}}\cdot \frac{r_{2}}{r_{1}}\right)\right]\frac{1}{\left(r_{2}-r_{1}\right)}$$

As an estimate for the maximum applicable range r_E_ we have replaced r_2_, A_2_ in this equation by r_E_, A_E_ and resolved for r_E_ .Under the assumption for the amplitude A_E_ at r_E_ to be at least twice the noise level, which might be different for every measurement environment, we have calculated values between 3 m and 5.5 m for our experimental setups [6,7]. The amplitudes for transducers installed after concreting are larger, probably because the special expanding grout used guarantees perfect coupling.

## 3. Short Notes on Ultrasonic Transmission Experiments

### 3.1. Wave Propagation in Concrete

Piezotransducers induce elastic waves into a concrete body. The propagation of these wave fields depends on the transducer, signal type and frequency as well as the body’s material, and geometry. In general, three types of waves are generated: Surface (Rayleigh) waves, which are slightly less important in our case, as we are using embedded transducers, and two types of body waves: compressional and shear. In a homogeneous linear elastic material the velocities of these waves (c_p_, c_s_) depend just on a few parameters: Young’s modulus E, shear modulus G, Poisson’s ratio ν, and density ρ:

(2) $$c_{p}=\sqrt{\frac{E(1-\text{υ})}{\text{ρ}(1+\text{ν})(1-2\text{ν})}}$$

and:

(3) $$c_{s}=\sqrt{\frac{G}{\text{ρ}}}$$

respectively.

In reality concrete is neither homogeneous nor fully linear elastic. In addition, ultrasonic waves are subject to reflections at internal boundaries or scattering at small (similar or smaller than the wavelength used) objects as aggregates, small cracks or reinforcement rebars. At the same time the amplitudes of the ultrasonic waves are affected by geometrical and intrinsical (material related) attenuation. From literature it is known that the following factors (and potentially also other) have an influence on ultrasonic velocities and the amplitudes:

- Concrete type and compressive strength ([8,9,10]);
- Stress ([11,12,13,14,15]);
- Temperature ([7,9,10,16,17]);
- Moisture ([18,19,20]);
- Degradation (microcracking) ([13,21,22,23]).

**Figure 9:**
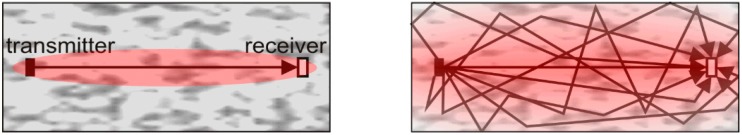
Sketch of ray paths (black lines)and area of influence (red) for the direct wave (**left**) and the full signal including coda (**right**).

Traditional time of flight measurements are considering direct waves only (first arrival at the receiver in most cases). The area of influence is limited to a narrow band (“first Fresnel zone”) between transmitter and receiver (Figure 9 left).

Lots of other waves, which arrive much later at the receiver, have undergone reflections and scattering, potentially even wave type conversion. It is quite often difficult to evaluate these arrivals separately. However, they contain useful information as they are covering larger areas of the concrete body (Figure 9 right) and are more sensible to velocity changes due to the longer travel paths.

### 3.2. Data Evaluation

The traditional way to interpret transmission data (“time of flight”) is described in the corresponding standards on ultrasonic pulse velocity measurements (see e.g., [10,24]). Picking of the first arrival travel times is often done manually. A lot of different automatic picking algorithms have been proposed from simple threshold pickers to more sophisticated ones based on statistical criteria. Based on our experience we prefer the Aikake Information Criterion (AIC) picker as described in [25]. This picker has been successfully used in many of our applications (e.g., [5]). Main disadvantage of the time of flight method is that changes in the medium under investigation may result in very small changes of the arrival times which are hard to detect. In addition, we would need a dense network of transducers in a construction to cover the entire volume by the relatively narrow Fresnel zones.

Recently methods have been introduced to ultrasound investigations in concrete, which are using the entire signal and not just the first arrival. Online monitoring systems can benefit from the use of correlation calculations (of a time series measured during/after load/damage against a reference one). Some more details and preliminary results have been published in [26]. For quantitative evaluation a novel method called coda wave interferometry may be used, which is able to calculate velocity changes from ultrasonic data with a sensitivity of about 2 × 10^−5^ [27]. The method can be expanded to tomographic imaging applications. So far, only a few applications to concrete have been reported [26,27,28,29].

## 4. Application Examples

### 4.1. Monitoring of Load Changes

**Figure 10:**
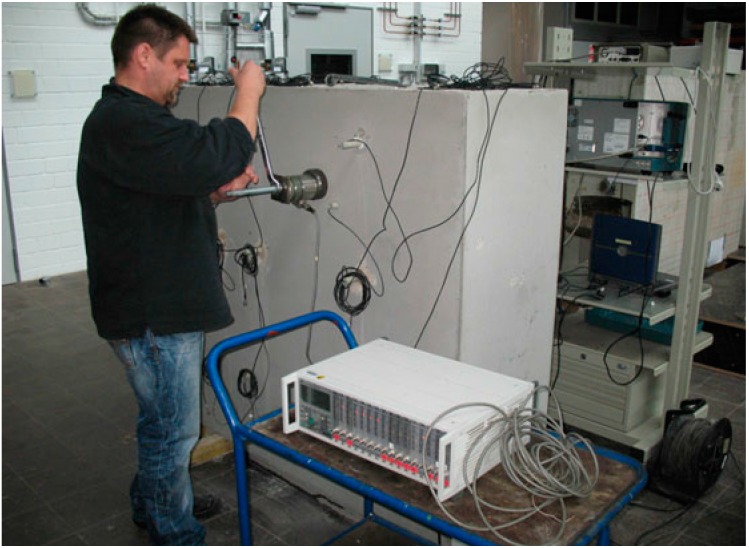
Concrete specimen GK32 with embedded transducers and load application system. From [26].

A 1.5 × 1.5 × 1.5 m^3^ concrete block (“GK32”) has been cast in the BAM labs for various tests of the embedded ultrasonic transducers (Figure 10). The lower half contains a certain amount of reinforcement, the upper one is unreinforced. A total number of 18 ultrasonic transducers have been embedded, partly during partly after casting the block. Just ten of those have been used for a load monitoring experiment due to limitation of the available data acquisition equipment. The experiment has already been described in more detail in [26]. A two channel multiplexer had connected the transducers to an ultrasonic transmitter (rectangle, 50 kHz) or data recording system, respectively. All 90 transducer combinations could be interrogated within seconds or minutes, depending on the number of repetitions. In the upper half of the concrete block a hole was drilled to insert a thread bolt. Some of the transducers have been just a few cm away from the center of the load, some almost 1 m. Nuts, 10 × 10 cm^2^ load distributing plates and a piezo load cell provided a way to introduce localized compressional stress in a controlled, repeatable way. Direction of the main compressional load is perpendicular to the front surface shown in Figure 10. However, stresses parallel to the front face are generated as well. Load steps of 5 or 10 kN were applied in various cycles up to a maximum load between 20 and 100 kN, more than one order of magnitude below the compressive strength of the concrete.

**Figure 11:**
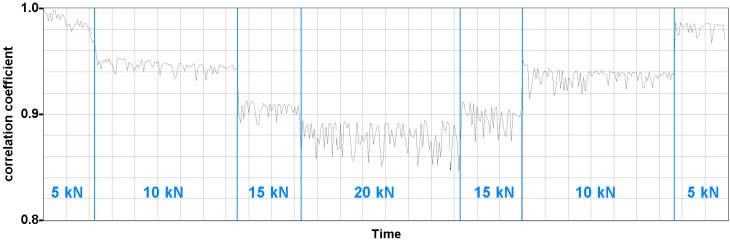
Correlation coefficient of 5 ms time series (reference: measurement at zero load) of ultrasonic signals measured by embedded transducers in a concrete block under small local compressional load. Line represents very dense consecutive measurements. From [26].

The applied loads, even very small ones, had a clear influence on the ultrasonic signals. A simple but valuable tool to provide a measure for the change is calculating the correlation coefficient between a reference measurement (here: zero load) and all consecutive measurement under various load conditions. Figure 11 shows the development of the correlations coefficient for transmission data between two embedded transducers close to the loading point. Both transducers have the same embedment depth (seen from the front face). Thus, direct waves are traveling perpendicular to the main load direction, but later parts of the signals (reflections, scattering) contain also information from different propagation directions.

Even small load changes of 5 kN can clearly be seen, even in the presence of noise. Tomographic coda wave evaluation of the entire embedded transducer data set showed that the area of significant influence of the load is limited to about 0.3 m away from the loading point [26].

### 4.2. Acoustic Emission

The acoustic emission (AE) technique monitors acoustic waves produced by newly developing micro cracks, opening and closure of existing cracks, friction, *etc*., all of which are caused by internal stress variations [30]. Based on techniques used in seismology the source of the acoustic emission can be localized. Traditionally the sensors used to detect AE events are arranged on the surface of the monitored element. The distance between them is restricted by the attenuation of the waves within the material. The frequency spectrum of the acoustic events in concrete is below 200 kHz [31]. If a set of embedded transducers would be able to perform passive (AE, localization of active cracks) and active measurements (determination of changes of velocity/attenuation/material parameters) at the same time, this would be a great step forward for structural health monitoring of concrete structures.

**Figure 12:**
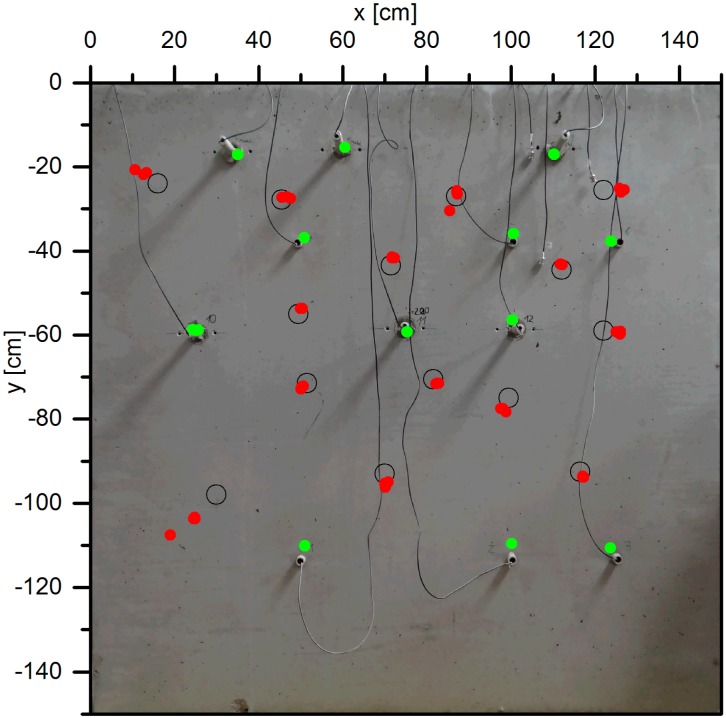
Location of acoustic emissions monitored with embedded ultrasonic transducers. Green marks: events localized by AE within the specimen (sources: one of the embedded transducers). Red marks: event localized by AE on the specimen’s surface (source: pencil breaks in black circles).

Laboratory tests on the use of the embedded transducers for AE were conducted using a similar specimen as discussed in Section 4.1 (“GK16”). All twelve embedded transducers were used with an AMSY6 recording system (Vallen Systeme GmbH, Icking, Germany) to detect acoustic emission events within the specimen and on its surface. All following measurements have been repeated three times. In a first step, the embedded transducers were used one at a time as artificial acoustic emission sources by sending a voltage pulse to each transducer successively. The average velocity of the compressional waves, measured during this automatic sensor test, was around 4500 m/s. Figure 12 shows the corresponding event localizations (green dots) calculated by the location processor option “Planar, plane” of the software Vallen VisualAE. They are coincident with the transducer locations.

In a second set of experiments pencil breaks on the specimen’s surface (a commonly used test for AE systems according to ASTM E976) have been used. Again, the experiments have been repeated three times at points marked by circles in Figure 12 and the events have been recorded and evaluated using the AMSY6 system. The localization (red dots in Figure 12) in areas with good transducer coverage lies on the mark ±3 cm. Those events outside the transducer array were not located accurately.

The experiment shows that the joint use of the embedded transducers in active and passive measurements is possible. The source needs to be within the sensor network to allow for an accurate localization. Future research will focus on the appropriate distance between the receivers for detecting and localizing acoustic emissions of different frequency and energy.

### 4.3. Time Reversal Experiment

This application example illustrates the use of the embedded transducers for model experiments in geophysical research. In geophysics, the validation of new approaches for measurement techniques or data evaluation is quite often limited to simulations as the real subsurface (especially in exploration when talking about several kilometers depth) can never fully be explored. So full scale experiments often lack of ground truth. Scaled model experiments might be of help.

**Figure 13:**
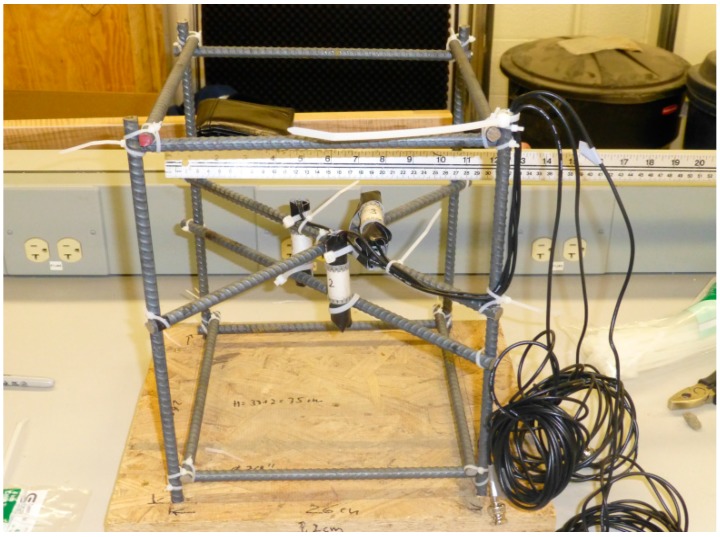
Setup of the three embedded transducers on the time reversal model experiment before concreting of the model, with permission from [33].

**Figure 14:**
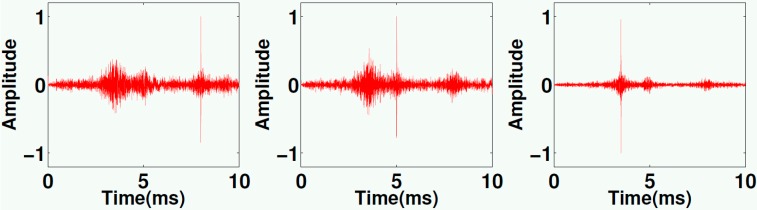
Backpropagated signals recorded at the three embedded transducers. Note the sharp peaks at specific times for each transducers reflecting perfectly the original time and place of the acoustic events, with permission from [33].

Time reversal is a technique to backproject elastic waves, which have been generated by events (e.g., cracks) inside a medium and registered by some sensors inside the medium as well or at the surface, numerically or physically back to their source point. Very few sensors are required for scattering media. In fact, the signals at the sensor, which have started as a spike at the source but then have been reflected, scattered, attenuated and dispersed on the way, can be focused back to the source point (as a perfect spike under ideal conditions). Recently a method to improve the results using deconvolution was proposed [32]. The transducers described in this paper have been used to verify the benefits of this method in case of multiple events overlapping in time [33]. For this purpose three transducers were embedded in a small concrete body (Figure 13) and served as ultrasonic sources (mimicking real acoustic emissions) in the first step. The data recorded at a single external receiver were then processed by the technology proposed in [32] and re-emitted by the external transducer into the model. Our embedded transducers now served as receivers. According to theory energy should focus at the position of a specific transducer at the time corresponding to the original event. This results in distinct sharp peaks in the recorded data (Figure 14). They correspond to the original events in time and shape. It could be shown, that the processed data focused much better in time and space at the transducers positions compared to unprocessed data. This experiment using our new transducers has definitively helped to prove the concept of using deconvolution in time reversal experiments, which would have been difficult under field conditions (events in some km depth) or doubted by practitioners if just done numerically. Details of the methodology, the experiment and the implications of the results are discussed in [33].

### 4.4. Other Applications

The embedded transducers have been applied in two other fields of applications so far. In several experiments we have used them to instrument concrete lab samples (typical size 15 × 15 × 40 cm^3^). These samples have been put into climate chambers or CDF test devices to evaluate the influence of temperature [16] and moisture on ultrasonic data. This evaluation is required in real world monitoring application as the influence of loads or deterioration is often covered by environmental influences. Instrumented lab samples may also be used in CDF test for freeze thaw resistance evaluations. The measurement of ultrasonic pulse velocity is already part of these evaluations, but the samples have to be removed from the CDF test chambers so far. Embedded transducers would make these tests much easier.

We have started to use the embedded transducers in full scale monitoring applications as well. An existing, degraded bridge in Turkey is monitored since more than two years by a set of eight transducers. A new bridge in Poland has been equipped with eight transducers before concreting. The hardening of the concrete could be monitored during the first 28 days. Load experiments (a requirement in Poland) will follow.

## 5. Conclusions

The ultrasonic transducers developed for embedment in concrete have shown to be valuable tools for various tasks in structural monitoring. They met our expectations in frequency (around 60 kHz), directivity (almost circular around the main axis) and range (at least three meters). The transducers proved to be very robust. We have developed deployment systems for existing and newly built structures. Early versions are now embedded and used in lab samples and real structures for a few years and are still fully operational.

We have shown that the transducers are useful in a lot of applications. They can be used for active transmission experiments as well as for collecting passive acoustic emission data. They proved to be useful in lab samples, scale experiments and real world monitoring systems. Load changes can be detected and localized as well as environmental influences (temperature, moisture) and various degradation mechanisms. In connection with novel interpretation tools as coda wave interferometry we have very sensible methods for the detection of changes in concrete at hand.

The embedment in concrete has various advantages: The coupling to the concrete is constant, transducers at lab samples have not to be removed before putting them into climate chambers, chemical baths or similar and installations are more vandal proof and less accident prone on real constructions.
